# Formulation and Characterization of Solid Dispersion Prepared by Hot Melt Mixing: A Fast Screening Approach for Polymer Selection

**DOI:** 10.1155/2014/105382

**Published:** 2014-03-12

**Authors:** Arno A. Enose, Priya K. Dasan, H. Sivaramakrishnan, Sanket M. Shah

**Affiliations:** ^1^Piramal Enterprise Limited, 1 Nirlon Knowledge Park, Goregaon East, Mumbai, Maharashtra 400063, India; ^2^Materials Chemistry Division, SAS, VIT University, Tamil Nadu 632014, India; ^3^Department of Pharmaceutics, Bombay College of Pharmacy, Kalina, Mumbai, Maharashtra 400098, India

## Abstract

Solid dispersion is molecular dispersion of drug in a polymer matrix which leads to improved solubility and hence better bioavailability. Solvent evaporation technique was employed to prepare films of different combinations of polymers, plasticizer, and a modal drug sulindac to narrow down on a few polymer-plasticizer-sulindac combinations. The sulindac-polymer-plasticizer combination that was stable with good film forming properties was processed by hot melt mixing, a technique close to hot melt extrusion, to predict its behavior in a hot melt extrusion process. Hot melt mixing is not a substitute to hot melt extrusion but is an aid in predicting the formation of molecularly dispersed form of a given set of drug-polymer-plasticizer combination in a hot melt extrusion process. The formulations were characterized by advanced techniques like optical microscopy, differential scanning calorimetry, hot stage microscopy, dynamic vapor sorption, and X-ray diffraction. Subsequently, the best drug-polymer-plasticizer combination obtained by hot melt mixing was subjected to hot melt extrusion process to validate the usefulness of hot melt mixing as a predictive tool in hot melt extrusion process.

## 1. Introduction

Amorphization of drug increases the solubility of drug because of increased surface area and better ability of the solvent to wet the drug. Such a process can improve the bioavailability of drugs that are poorly soluble, namely, BCS class II and BCS class IV drugs [1–3]. For dissolution rate limited drug absorption, molecularly dispersed drug can improve dissolution and thus its absorption [4, 5]. Hot melt extrusion is a solvent free process that utilizes heat and pressure to disperse the drug molecularly in a given polymer-plasticizer combination [6–14]. The technique is similar to preparation of solid dispersion by melting method. Formulating a molecularly dispersed form of drug in a carrier matrix requires the optimum amount of drug along with polymer and plasticizer. In some instances a drug (Ibuprofen) itself acts as a plasticizer thus limiting the amount of plasticizer that can be added [8, 15]. Film casting method can be used as preliminary screening technique to determine the right amount of drug-polymer-plasticizer combination that can yield molecularly dispersed drug [16, 17]. The polymer and plasticizer are solubilized in a solvent and applied on a glass plate using a casting device. The film upon drying is visually and microscopically observed to determine any drug precipitation. DSC and X-ray diffraction are other advanced analytical techniques that can be used to determine the amorphization of drug in polymer based on changes in thermal behavior and crystallinity of drug. Hot melt mixing is another screening technique, described in the current study, which can be used in selection of possible drug-polymer-plasticizer combination for employing in hot melt extrusion (HME) process.

Sulindac (SUL) is a nonsteroidal anti-inflammatory agent belonging to BCS class II, the absorption of which is dissolution rate limited. Solubility of SUL is 56 *μ*M in water and the solubility increases with increasing pH (up to 15 mM at pH 7.4). Amorphization of SUL can increase its solubilization in the GIT resulting in increased concentration of SUL at the site of absorption.

**Scheme 1:**
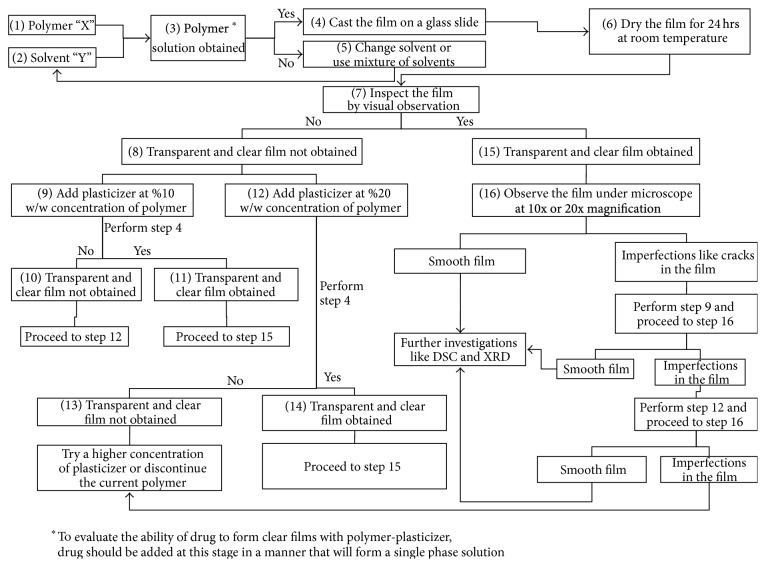
Approach for screening and selection of polymer and its combination with plasticizer.

In the current investigation, a systematic approach is described that can be used for development of hot melt mix/extrudates of drugs with varying physical properties (Scheme 1). Many of the new chemical entities have good activity but lack good physicochemical properties. Low solubility, low absorption, and/or both are the major factors responsible for failure of a new chemical entity. The former issue of solubility can be taken care of by formulating molecularly dispersed drug in form of solid dispersion. In development of HME a very large quantity of drug is required which is a limitation in new drug discovery process. Here we propose a systematic approach for determining the right combination of drug-polymer-plasticizer using minimal quantity of drug. After initial film forming studies, a process (hot melt mixing) that tries to mimic the HME process was developed to formulate a solid solution of the drug in polymer. The amorphization of SUL was confirmed by optical microscopy, differential scanning calorimetry (DSC), X-ray diffraction, hot stage microscopy [18], and dynamic vapor sorption [6, 10, 11, 19–22]. The solid solution/dispersion was evaluated for drug content, purity, solid state stability by DSC, and increase in solubility at different pH (water, pH 1.2, pH 4.5, pH 6.8, and pH 7.4) [23]. The solubility of SUL was increased by many folds in all the media tested indicating better dissolution in the GIT. Finally the drug-polymer-plasticizer combination screened by film casting method and hot melt mixing was subjected to HME to confirm the usability of hot melt mixing as a tool for predicting behavior of drug-polymer-plasticizer combination in a HME process.

## 2. Materials and Methods

### 2.1. Materials

Glyceryl behenate (GB) and hydroxyl propyl methyl cellulose (HPMC) were obtained from Colorcon India Pvt. Ltd., Mumbai, India. Lauroyl macrogolglycerides (LM) and stearoyl macrogolglycerides (SM) were obtained from Gattefosse India Pvt. Ltd., Mumbai, India. Polyvinyl caprolactam-polyvinyl acetate-polyethylene glycol (57 : 30 : 13) graft copolymer (PPP), copovidone (CO), povidone (PO), poloxamer 407 (P407), and glycerol polyethylene glycol oxystearate (C 40) were obtained from BASF India Ltd., Mumbai, India. Glyceryl monostearate (GM), D-alpha-tocopherol polyethylene glycol 1000 succinate (TPGS), hydroxypropyl cellulose (HPC), chitosan (CHI), PEG1000, and triethyl citrate (TEC) were obtained from Sasol, Cognis Corporation, Aqualon Hercules, Sigma Life Sciences, and Merck and Aldrich Chemistry, respectively. Sulindac was obtained from Piramal Healthcare Limited, Mumbai, India. All solvents and reagents used were of analytical grade unless otherwise specified.

### 2.2. Preparation of Films

Experiments were carried out to determine the right combination of polymer-plasticizer concentration that could yield clear and transparent films (approximate diameter ~2 cm). The polymer and plasticizer in different ratios were dissolved in solvent/mixture of solvents (as described in Scheme 1), followed by film formation on a glass surface by evaporation of solvent at 24°C for 24 hours. A volatile solvent was preferred over water or high boiling point solvents as the solvent would take longer time to evaporate and there is a possibility of incomplete drying of the film. In case of chitosan, after air drying for 24 hours at 25°C, the film was further dried on a hot plate (C-MAG HS7, IKA India Pvt. Ltd., India) maintained at 100°C for 30 minutes. The plasticizers were tested at two levels, namely, 10% w/w and 20% w/w of the polymer concentration. The various plasticizers and the combination with different polymers are shown in Table 1. For HPMC and chitosan, the plasticizers were dissolved in ethanol and mixed with polymer solution. For other polymers, chloroform was used as the solvent. After choosing the polymer-plasticizer combination based on visual and optical microscopic analysis, sulindac (dissolved in ethanol at 10 mg/mL) was added to the polymer-plasticizer solution at three different concentrations of 10% w/w, 20% w/w, and 50% w/w of polymer. The dried film was then observed microscopically to observe any grittiness or drug precipitation.

### 2.3. Visual and Microscopic Analysis of Films

The films after complete drying were observed visually for smoothness and absence of any cracks or drug precipitate. For the purpose of optical microscopy (Leica Microsystems, Germany), the films were observed in crosspolarized light incident at 90° at magnification of 40x and recorded digitally (color video camera, JVC, India) using Leica QWin V3 software. In case of drug containing films, the precipitate of drug was observed as a sign of crystalline substance present at the concentration tested for drug-polymer-plasticizer.

### 2.4. Formulation of SUL Solid Dispersion by Hot Melt Mixing Mimicking HME

In order to mimic the process of HME, a technique was developed wherein drug, polymer, and plasticizer (Table 2) in a fixed ratio (as obtained from initial screening studies) were geometrically mixed in a ground glass test tube. The mixture was heated in liquid paraffin contained in a beaker which was heated on a hot plate (C-MAG HS7, IKA India Pvt. Ltd., India) maintained at 170°C with concomitant mixing. The hot mass obtained was immediately removed, allowed to cool, and stored in glass vials at 2–8°C. Microscopic analysis was performed for solid dispersions prepared by hot melt mixing, physical mixture of polymer-plasticizer, and physical mixture of drug-polymer-plasticizer, the last two prepared by simple geometric mixing. The % yield and % content of SUL was determined by UV spectroscopy at 327 nm [24].

### 2.5. Thermal Analysis of SUL Solid Dispersion

To confirm the presence of molecularly dispersed form of SUL in formulations described in Table 2, a detailed thermal characterization was performed using DSC (Diamond DSC, Perkin-Elmer Inc., USA). Approximately 2 mg of sample was placed in a hermetically sealed aluminum pan (50 *μ*L) with a pinhole at a nitrogen purge of 20 mL/min, with a scan rate of 10°C/min from 40°C to 200°C, and analyzed by Pyris series-Diamond DSC software.

### 2.6. Hot Stage Microscopy Study

A small quantity (~1 mg) of SUL and formulations described in Table 2 was sandwiched between two glass cover slips (W16G, Linkam, UK) placed inside a heating stage (hot stage, HFS91, Linkam, UK). The stage was placed under a polarized microscope (Leica Microsystems, Germany) and the images were recorded using a digital color video camera (TK-C14803, JVC, Japan) and the images were processed using Linksys 32 software (Germany). The sample was heated from 25°C to 300°C at a rate of 20°C/min and images were taken after fixed internals to visualize the thermal changes that occur during heating.

### 2.7. X-Ray Diffraction Study

About (~3 mg) of SUL and different formulations was assessed by X-ray powder diffraction (D-8 advanced type Bruker instrument). The samples were exposed to CuK*α* radiation under 40 kV and 40 mA over the 2-theta range from 3° to 40°C at increments of 0.25°/minute.

### 2.8. Solubility Study

Solubility of SUL and its formulations was determined in water, pH 1.2, pH 4.5, pH 6.8, and pH 7.4. The buffers were prepared as described in USP (USP 30 NF 25, Volume 1). Briefly SUL (15 mg) or its formulations (15 mg equivalent) were placed in a glass vial followed by solvent (3 mL) addition and cyclomixing for 30 sec (Vortex-Genie 2, Scientific Industries, USA). The glass vials were then placed in temperature control bath (BS-21, Lab Companion, Malaysia) at 37°C for a total duration of 24 hours. Intermittently, the vials were checked for complete solubility of SUL and if required more amounts of SUL or its formulations were added. After 24 hours, the solution was filtered through 0.45 *μ*m filter (hydrophilic PVDF, Millex-HV, Millipore, India) and analyzed by UV spectroscopy (Shimadzu, Pharmaspec UV-1700) at a wavelength of 327 nm and solubility determined.

### 2.9. Solid State Stability

Solid state stability of the formulations was carried out in stability chambers maintained at different temperatures and durations (25°C/65% RH for 15 days, 40°C/75% RH for 15 days, 50°C for 3 days, and 100°C for 3 days). The formulations evaluated were SUL + PPP + PEG1000, SUL + PPP + TEC, SUL + HPC + PEG1000, SUL + HPC + TEC, SUL + HPMC + PEG1000, and SUL + HPMC + TEC. For the purpose of sample preparation the required amount of SUL or its formulations was placed in clear glass vials, closed with rubber closure, and sealed with aluminium cap. The samples after predetermined time were analyzed by DSC and X-ray diffraction for their thermal stability and stability towards formation of drug crystals.

### 2.10. Dynamic Vapor Sorption

Based on the solubility and solid state stability data, few formulations listed in Table 2 were further evaluated for their water absorption behavior in controlled humidity conditions. A known amount of the solid dispersions of SUL was weighed and kept in DVS (DVS Advantage, SMS, UK) with controlled temperature and pressure chamber. The experiment was programmed for sorption for a period of 3 hours in each humidity condition from 0%, 30, 60, and 90% and constant temperature of 25 degrees. The change in mass was monitored with varying humidity conditions to understand the effect of moisture on solid dispersions of SUL prepared by hot melt mixing. This is another characterization technique that can be used to understand the effect of moisture on the possible transformation of a system from amorphous to crystalline state when subjected to increased humidity.

### 2.11. Hot Melt Extrusion

After the screening of different drug-polymer-plasticizer combinations, the optimal combination of the three that provided the best amorphization of SUL and improved solubility and better stability was selected for performing extrusion on a lab scale hot melt extruder (Thermo Scientific, Pharma II hot melt extruder). A combination of SUL (20% w/w of PPP), PPP, and PEG1000 (20% w/w of PPP) was selected based on its solubility, solid state stability, and physical characterization. The process parameters were as set at torque of 30%–40%, with a screw speed of 100 rpm; process temperature was programmed in an increasing mode starting at 115°C, increased to 130°C in second phase and the final phase maintained at 145°C in the different heating zones. The obtained hot melt extrudates were characterized by DSC and X-ray diffraction studies to confirm the presence of molecularly dispersed SUL.

## 3. Results

### 3.1. Film Analysis

**Figure 1:**
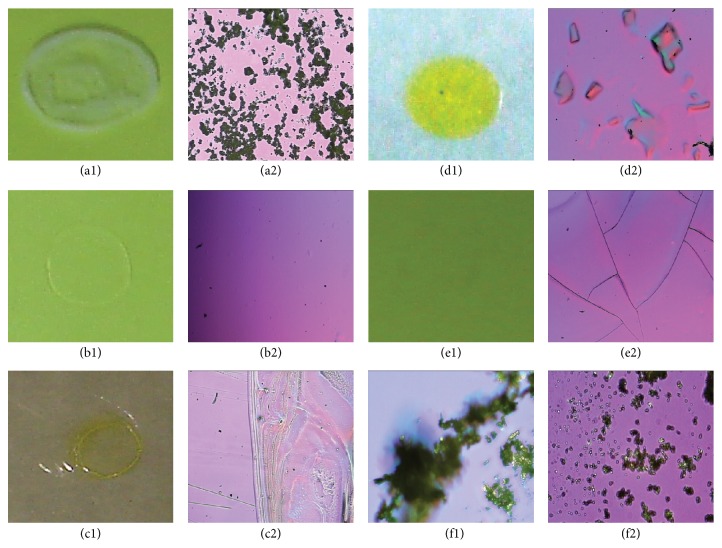
Images of films obtained after evaporation of solvent after 24 hours. All the images on the Left are taken by a high resolution digital camera and the images on the right are taken with an optical microscope using a crosspolarized light at 90°. Film of glyceryl monostearate with 20% w/w TEC as observed by visual (a1) and optical microscopy (a2), film of hydroxypropyl methyl cellulose with 20% w/w TEC as observed by visual (b1) and optical microscopy (b2), film of hydroxyl propyl cellulose with 20% w/w PEG1000 and 20% w/w sulindac as observed by visual (c1) and optical microscopy (c2), film of chitosan with 20% w/w glycerol polyethylene glycol oxystearate and 50% w/w sulindac as observed by visual (d1) and optical microscopy (d2), film of povidone with 20% w/w C 40 as observed by visual (e1) and optical microscopy (e2), crystals of sulindac as observed under bight field (f1) and polarized light at 90° (f2) by optical microscope.

The visual and optical analysis of the films using crosspolarized light at 90° are shown in Figure 1. Representative examples for selection of drug-polymer-plasticizer combination are shown. GM with 20% TEC did not produce clear and smooth films as evident from visual analysis (Figure 1(a1)). The grittiness of the film was also confirmed by optical microscopy (Figure 1(a2)). On the other hand, in case of HPMC, a clear and transparent film was obtained with all the plasticizers tested; a representative example with 20% TEC as observed visually (Figure 1(b1)) and optical microscopy (Figure 1(b2)). After initial screening the polymers selected for testing with drug were HPMC, HPC, CO, PO, CHI, and PPP. The plasticizers that were selected for further analysis were TEC and PEG1000 due to superiority of the films obtained. SUL was added at three different concentrations of 10% w/w, 20% w/w, and 50% w/w of the polymer amount. A representative image of HPC, 20% w/w TEC, and 20% w/w SUL is shown in Figure 1(c1) (visual) and Figure 1(c2) (optical microscopy). Microscopic analysis of the film containing chitosan, 20% w/w C 40, and 50% w/w SUL showed deposition of crystals after drying as evident in Figures 1(d1) and 1(d2). Films containing povidone with 20% w/w C 40 (Figures 1(e1) and 1(e2)) showed presence of cracks upon microscopic analysis indicating poor film properties.

### 3.2. Microscopic Characterization of SUL Solid Dispersion Prepared by Hot Melt Mixing

**Figure 2:**
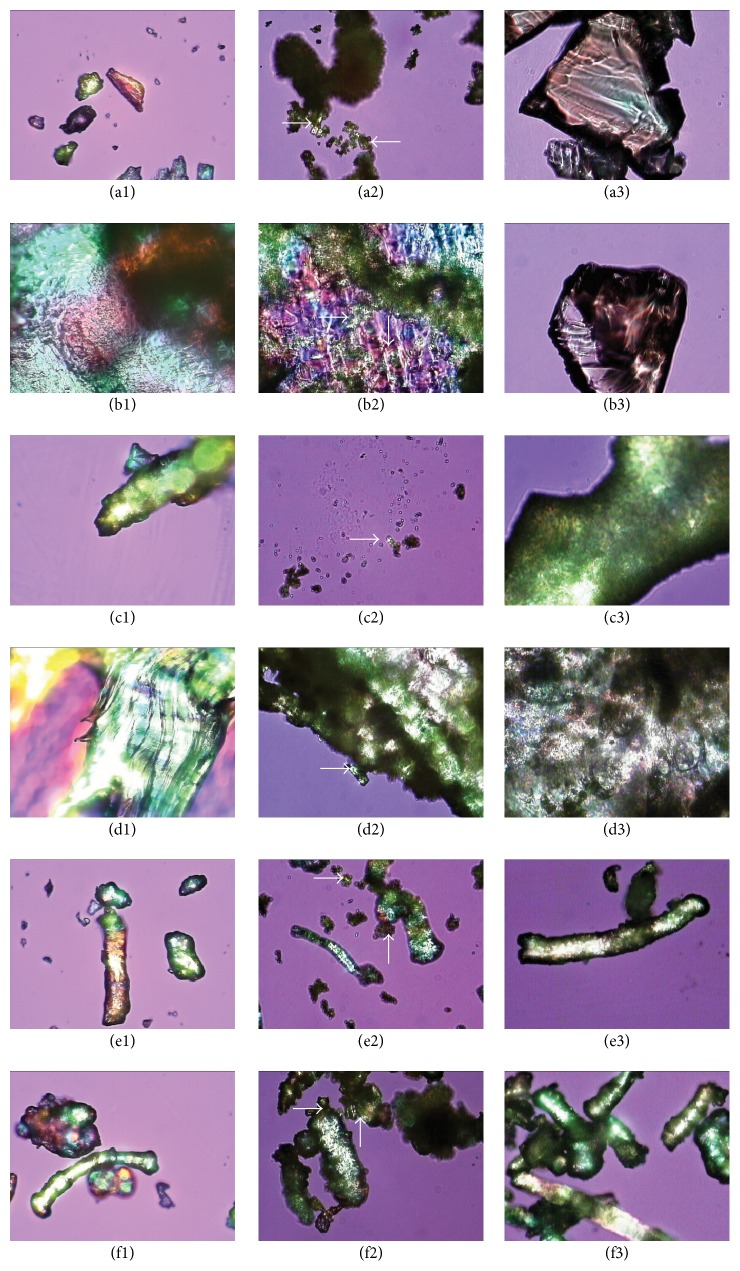
Optical microscopic image (40x magnification) using crosspolarized light at 90° for batches described in Table 2. Image of physical mixture of PPP and 20% w/w PEG1000 (a1); physical mixture of PPP, 20% w/w PEG1000, and 20% w/w SUL (a2); hot melt mix of PPP, 20% w/w PEG1000, and 20% w/w SUL (a3); physical mixture of PPP and 20% w/w TEC (b1); physical mixture of PPP, 20% w/w TEC, and 20% w/w SUL (b2); hot melt mix of PPP, 20% TEC, and 20% w/w SUL (b3); physical mixture of HPC and 20% w/w PEG1000 (c1); physical mixture of HPC, 20% w/w PEG1000, and 20% w/w SUL (c2); hot melt mix of HPC, 20% w/w PEG1000, and 20% w/w SUL (c3); physical mixture of HPC and 20% w/w TEC (d1); physical mixture of HPC, 20% w/w TEC, and 20% w/w SUL (d2); hot melt mix of HPC, 20% w/w TEC, and 20% w/w SUL (d3); physical mixture of HPMC and 20% PEG1000 (e1); physical mixture of HPMC, 20% w/w PEG1000 and 20% w/w SUL (e2); hot melt mix of HPMC, 20% w/w PEG1000, and 20% w/w SUL (e3); physical mixture of HPMC and 20% TEC (f1); physical mixture of HPMC, 20% w/w TEC, and 20% SUL (f2); hot melt mix of HPMC, 20% w/w TEC, and 20% SUL (f3).

A bright field and polarized light microscopic image of SUL are shown in Figures 1(f1) and 1(f2). Polarized light microscopic image of formulations described in Table 2 along with physical mixtures of drug-polymer-plasticizer in the same weight ratio are shown in Figure 2. The images on extreme left (Figures 2(a1), 2(b1), 2(c1), 2(d1), 2(e1), and 2(f1)) are of physical mixtures of polymer and 20% w/w plasticizer without SUL. The images at the centre (Figures 2(a2), 2(b2), 2(c2), 2(d2), 2(e2), and 2(f2)) are of physical mixtures of polymer, 20% w/w plasticizer, and 20% w/w SUL. The images on the extreme right (Figures 2(a3), 2(b3), 2(c3), 2(d3), 2(e3), and 2(f3)) are of the hot melt mix of polymer, 20% w/w plasticizer, and 20% w/w SUL. Physical mixture of polymer and plasticizer showed optical birefringence as evident in all the polymers evaluated. When SUL was added to the physical mixture of polymer and plasticizer, it adsorbed onto the surface of the polymer as showed by white arrow in Figure 2. Crystals of SUL were not observed on the surface of formulations prepared by hot melt mixing. Polarized microscopy confirmed that the drug-polymer-plasticizer has formed a one phase system that exhibits no property similar to individual optical properties of drug, polymer, or plasticizer.

### 3.3. Yield and Content of SUL Solid Dispersions

The % yield of SUL hot melt mix was determined (Table 2) to ascertain the losses incurred during the hot melt mixing process. The content of SUL in each formulation was determined to evaluate the correctness of the process. The % yield for all the formulations was close to 100% with minimal wastage. For formulation B, the yield was comparatively low due sticky nature of the drug-polymer-plasticizer dispersion. The % content of all the formulations was within limits indicating homogeneity and accuracy of the process.

### 3.4. Thermal Analysis of Formulations

**Figure 3:**
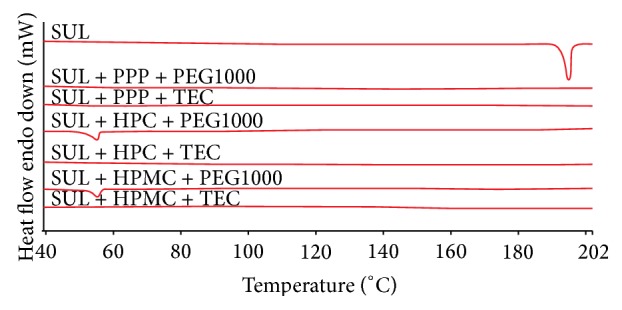
DSC thermograms of SUL and formulations described in Table 2 taken at heating rate of 10°C/min from 40°C to 200°C.

Thermal analysis by DSC for SUL showed a sharp endotherm at 188°C as observed in Figure 3. Solid dispersions of SUL prepared by hot melt mixing did not show any sharp endotherm from 40°C to 200°C. A sharp endotherm was observed in thermogram of SUL with peak at 188°C. In the formulations of SUL prepared by hot melt mixing, no endothermal peak for SUL was observed at 188°C or at a lower temperature suggesting possible molecular dispersion of SUL in polymer-plasticizer combination. DSC results confirm that SUL has been molecularly dispersed in the matrix of polymer and plasticizer. A broad endotherm at 56°C in case of combinations SUL + HPC + PEG1000 and SUL + HPMC + PEG100 is due the melting of PEG1000. A small dip in the baseline at around 80°C in SUL + PPP + PEG100 and SUL + PPP + TEC could be due to glass transition temperature of PPP at around 70°C. Formulations containing TEC as plasticizer, endothermic events were not observed.

### 3.5. Hot Stage Microscopic Study of Formulations

**Figure 4:**
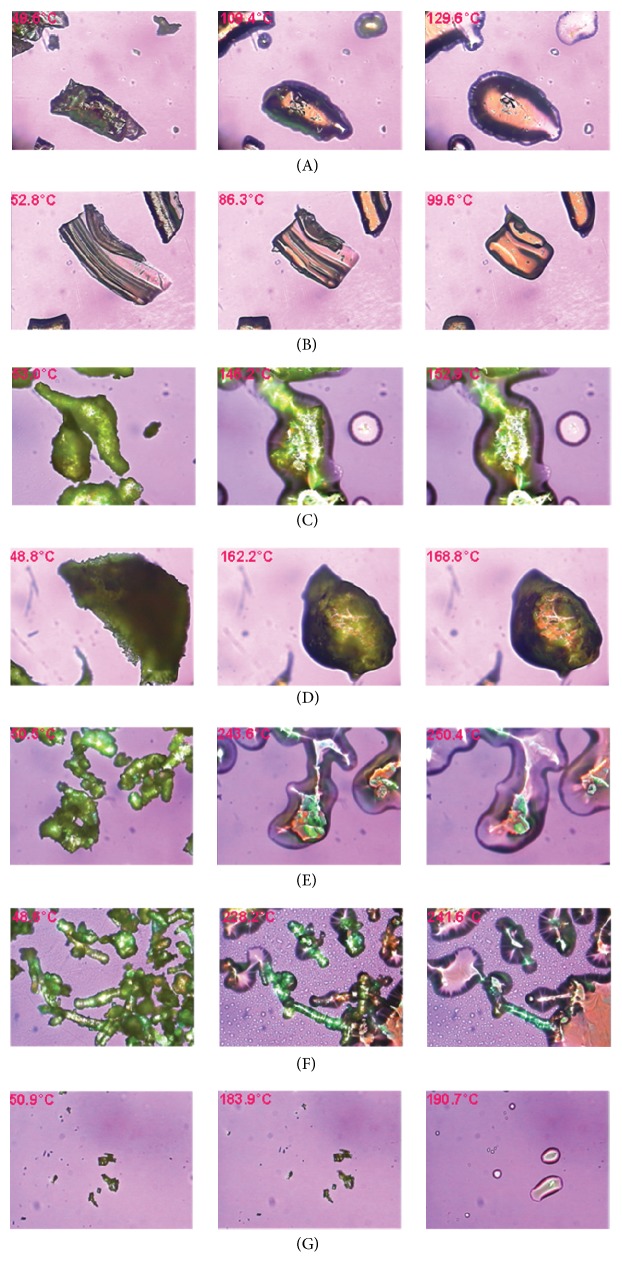
Hot stage microscopy study of formulations described in Table 2 ((A) to (F)) and SUL (G). The images on the extreme left are the initial images of all samples tested. The middle and the right image are the images where thermal changes are observed, especially melting. (A) and (B) observed at a magnification of 10x, (C) observed at 20x, (D) observed at 10x, (E) and (F) observed at 20x, and (G) observed at 40x magnification in polarized light.

The hot stage microscopy images are shown in Figure 4. SUL exhibited melting at 190.7°C (Figure 4(G)) which correlate well with the sharp endothermic transition observed at 188°C in DSC. No such thermal event was observed for formulations A to F between 185°C and 190°C. For Figure 4(A), with PEG1000 as the plasticizer, the thermal event started at 110°C whereas in Figure 4(B), with TEC as the plasticizer, the thermal event was shifted to 86°C. This behavior can be attributed to the glass transition temperature of PPP at 70°C. For Figures 4(C) and 4(D) containing HPC, with PEG1000 and TEC as the plasticizers, respectively, the melting initiated at 146°C and 162°C, respectively. For Figures 4(E) and 4(F) containing HPMC, with PEG1000 and TEC as the plasticizers, respectively, melting initiated at 243°C and 228°C, respectively. For SUL complete melting was observed in a span of 2°C after its initiation. For formulations of PPP, HPC, and HPMC there was a broad range of 20°C–30°C during which complete change of state was observed. Molecular dispersion of SUL in various polymers is confirmed as no specific thermal changes were observed at 188°C.

### 3.6. X-Ray Diffraction Study of Formulations

**Figure 5:**
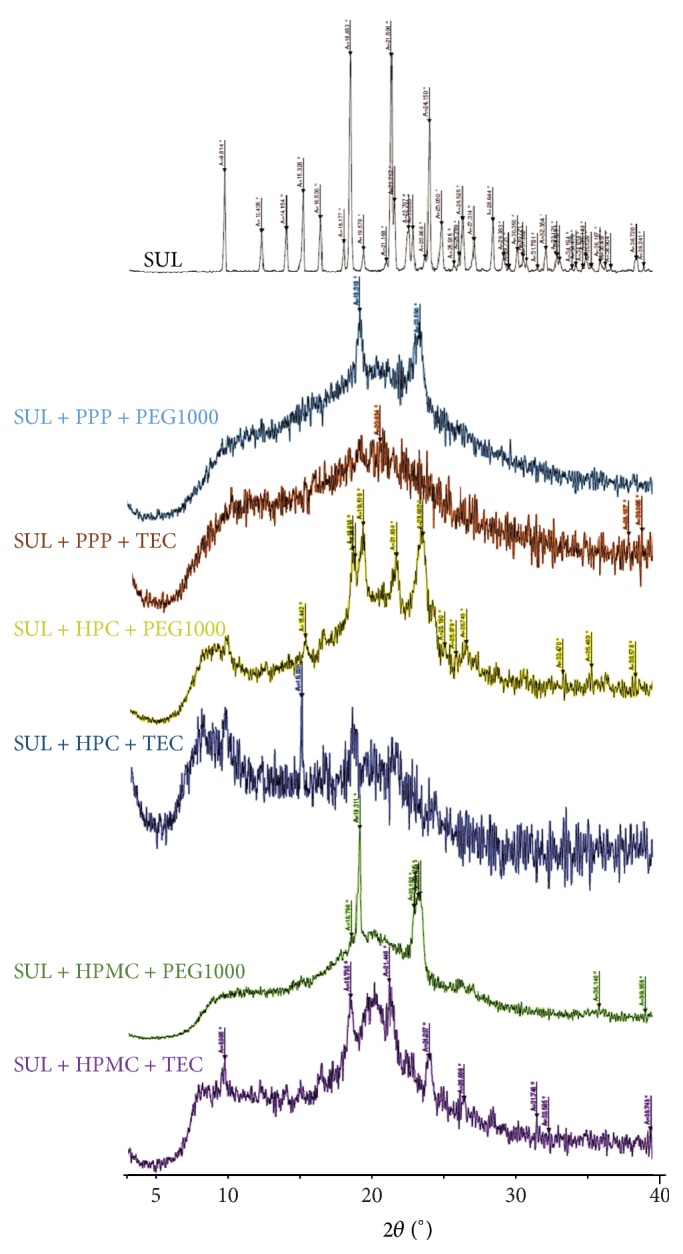
X-ray diffraction pattern of SUL and its various formulations described in Table 2.

As can be seen in Figure 5, the evidence of amorphization of SUL in different polymer-plasticizer combinations was evident as there was no sharp peak present in the X-ray diffraction pattern of many when compared to that of pure SUL. In case of SUL + HPMC + PEG1000 and SUL + HPMC + TEC there were a few sharp peaks evident but nevertheless the rest of the diffraction pattern was diffused as is the case with amorphous substances.

### 3.7. Solubility of SUL Formulations

**Figure 6:**
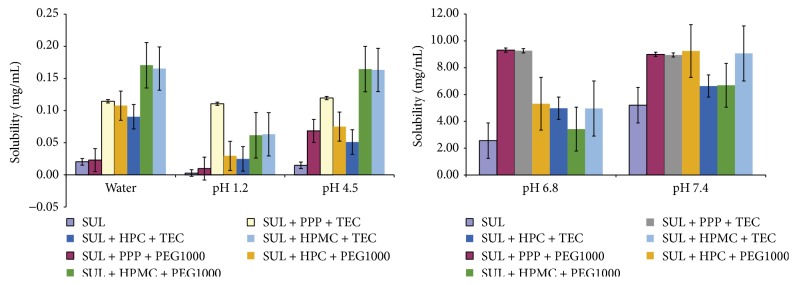
Solubility of sulindac and its formulations described in Table 2.

The solubility of SUL and its formulations is shown in Figure 6. SUL has a very low solubility in water, pH 1.2, and pH 4.5 due to its pKa of 4.7. A high solubility of SUL is expected above pH 4.7 due to formation of salt. At low pH value conditions of pH 1.2 and pH 4.5, formulations B, E, and F were superior. At basic pH value conditions of pH 6.8 and pH 7.4, formulations A, B, C, and F were superior. There was an 8-fold increase in the solubility of SUL in water and pH 4.5 for formulations E and F and up to 40-fold increase in solubility of SUL in pH 1.2 for formulation B. The solubility of SUL in pH 6.8 and pH 7.4 was high (pKa 4.7). Up to 4-fold increase in solubility of SUL in pH 6.8 for formulations A and B was observed. Up to 2-fold increase in solubility of SUL in pH 7.4 for formulation C was observed. The results confirm the increase in solubility of SUL due to its amorphization and thus increased surface area exposure to the solvent.

### 3.8. Solid State Stability

**Figure 7:**
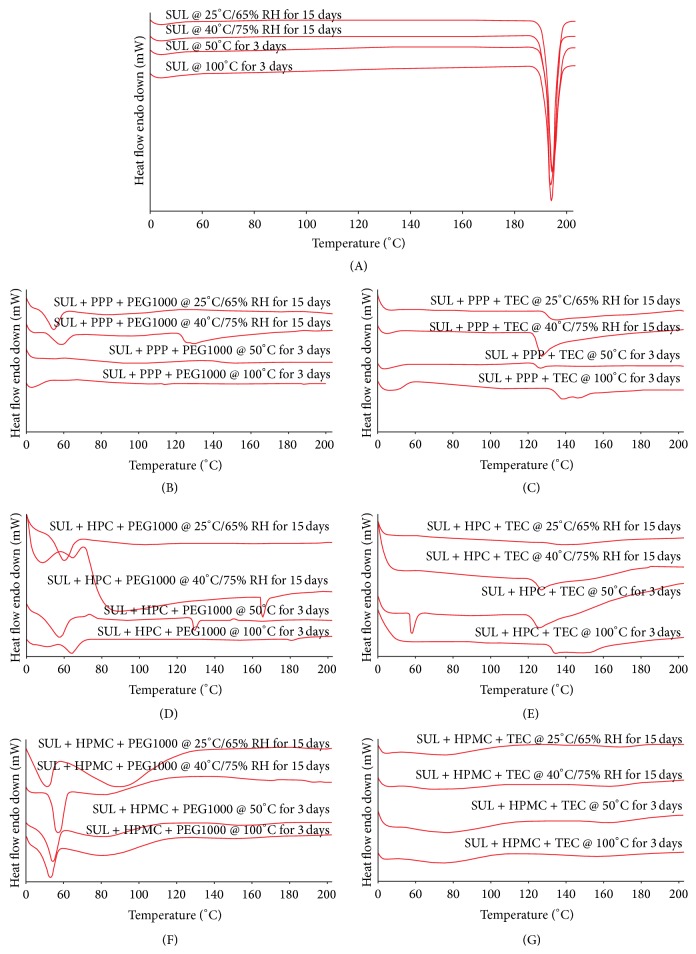
Solid state stability of different formulations described in Table 2 under accelerated conditions. The formulations evaluated were SUL (A), SUL + PPP + PEG1000 (B), SUL + PPP + TEC (C), SUL + HPC + PEG1000 (D), SUL + HPC + TEC (E), SUL + HPMC + PEG1000 (F), and SUL + HPMC + TEC (G). All the formulations were evaluated at 25°C/65% RH for 15 days, 40°C/75% RH for 15 days, 50°C for 3 days, and 100°C for 3 days.

Solid state stability results based on DSC (Figure 7) indicated superiority of formulations consisting of PPP and HPMC as the polymers. Amongst the plasticizers, PEG1000 was superior to TEC. In case of HPC as the polymer, drug expulsion was indicated at elevated temperatures based on an endothermic transition at around 160°C. In case of PPP and HPMC, there was no evidence of drug expulsion from DSC investigations.

### 3.9. Dynamic Vapor Sorption

**Figure 8:**
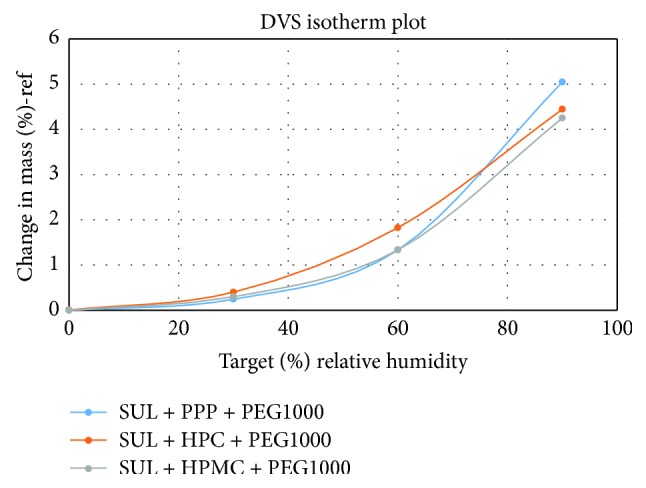
Dynamic vapor sorption plot of SUL + PPP + PEG1000, SUL + HPC + PEG1000, and SUL + HPMC + PEG1000.

As shown in Figure 8 all the three formulations evaluated, namely, SUL + PPP + PEG1000, SUL + HPC + PEG1000, and SUL + HPMC + PEG1000, showed expected behavior. The % change in weight was not more than 2% at 60% relative humidity and not more than 4% at 80% relative humidity. This is an indication that humidity will not play a crucial role in determining the amorphous nature of SUL in the formulation. The current combination of drug-polymer-plasticizer is not affected by increased humidity.

### 3.10. Hot Melt Extrusion

**Figure 9:**
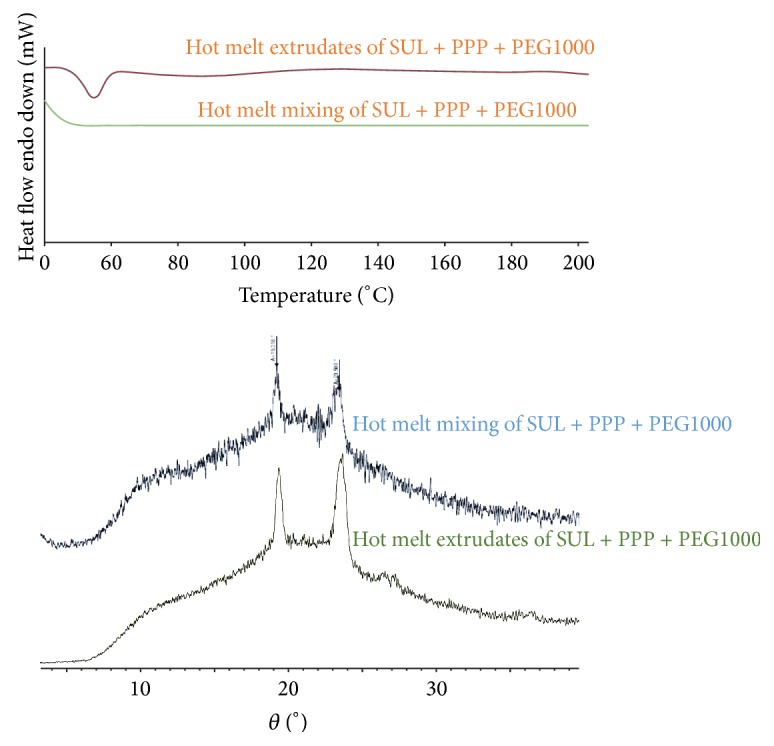
DSC and X-ray diffraction of hot melt extrudates prepared using SUL (20% w/w) and PPP and PEG1000 (20% w/w) using a mini lab extruder.

Based on the initial film screening technique followed by screening using hot melt mixing a final combination of drug-polymer-plasticizer was selected for hot melt extrusion. The formula consisted of SUL (20% w/w of polymer), PPP, and PEG1000 (20% w/w of polymer). To validate the claim of hot melt mixing as a preliminary tool for selecting right drug-polymer-plasticizer combination using minimal amount of drug, DSC and X-ray (Figure 9) diffraction studies were performed to ascertain the molecularly dispersed form of SUL in the hot melt extrudates of SUL-PPP-PEG1000. The DSC investigation showed similar thermal behavior of extrudates with that prepared by hot melt mixing. Also X-ray diffraction pattern pointed towards the amorphous nature of the drug in the extrudates comparable to the ones prepared by hot melt mixing.

## 4. Discussion

To formulate molecularly dispersed form of drug a streamlined approach for selecting the right drug-polymer-plasticizer based on film casting technique is described. Hot melt mixing mimicking hot melt extrusion is described to ascertain the possibility of forming solid dispersions using HME. Optical microscopic analysis of the polymer-plasticizer films revealed potential combinations that could be used for their combination with SUL. The polymer-plasticizer combination that gave a clear and smooth film was selected for screening with drug. C 40 did not give smooth films with most of the polymers. Films prepared from polymers (PPP, PO, CO, HPC, HPMC, and chitosan) using PEG1000 (20% w/w) and TEC (20% w/w) as plasticizers were chosen for further studies with sulindac due to their superiority in terms of visual appearance and optical microscopic analysis. The polymers studied for their feasibility in hot melt mixing process were PPP, HPC, and HPMC (these polymers formed superior films with sulindac) with PEG1000 (20% w/w) and TEC (20% w/w) as the plasticizers and the drug concentration fixed at 20% w/w. All the polymers that were tested with sulindac for their feasibility in hot melt mixing process gave uniform dispersion without presence of drug, polymer, or plasticizer pockets in the matrix. The dispersion of sulindac with PPP was visible as a solid solution whilst that with HPMC and HPC was a uniform dispersion. Dispersions containing TEC as a plasticizer had more binding strength and rigidity as compared to dispersions consisting of PEG1000 as a plasticizer. The technique developed for hot melt mixing gave the drug content of close to 100% indicating homogeneity and accuracy of the process. DSC analysis of the dispersions prepared by hot melt mixing process confirmed the presence of sulindac in molecularly dispersed form as no endothermic event was evident. Dispersions of HPC and HPMC containing PEG1000 as the plasticizer gave a broad endotherm at 56°C for PEG1000 suggesting possible separation of PEG1000 from the dispersion due to faster cooling rate of PEG1000 as compared to other constituents. Hot stage microscopy studies validated the results obtained by DSC. No endothermic event was observed at 188°C. All the thermal events that were observed were at temperatures other than the melting point of SUL. X-ray diffraction study of all the formulations confirmed the molecular dispersion of SUL in different polymers. The characteristic peaks of SUL were either absent or subdued in all the polymer-plasticizer tested. Solubility of sulindac was checked in water and buffers (pH 1.2 to 7.4) to ascertain the increase in solubility of sulindac in dispersion as compared to only sulindac. Promising results were obtained for all the dispersions tested as there was significant increase in solubility in water as well as in all buffers tested. There was an 8.5-fold increase in solubility for dispersion containing sulindac, HPMC, and TEC in water. Almost 40-fold increase in sulindac solubility at pH 1.2 was observed for dispersion containing sulindac, PPP, and TEC. An 11-fold increase in solubility at pH 4.5 was observed for dispersions containing sulindac, HPMC, and TEC or PEG1000. As the pKa of sulindac is 4.7, there is bound to be an increase in its solubility above this pH due to formation of salts. This was evident as there was a drastic change in solubility of sulindac in free form and or its conjugation in form of dispersion. At pH 6.8 there was an 8-fold increase in solubility for dispersion containing sulindac, PPP, and PEG1000 or TEC. At pH 7.4 there was a twofold increase in solubility for dispersion of sulindac with HPC and PEG1000 as well as HPMC and TEC. All the polymers tested showed a significant increase in solubility of sulindac with dispersions containing TEC as the plasticizer showing higher solubilization power. On further investigation it was confirmed that sulindac is soluble in TEC (liquid at room temperature) and thus can accommodate drug in its matrix leading to enhanced solubilization of sulindac. The solid state stability showed that the TEC as a plasticizer, although showing better solubilization potential of SUL, did not hold the drug in molecularly dispersed form upon subjecting it to stressed solid state stability studies. This was evident in DSC thermograms of combinations containing TEC as plasticizer. HPC did not produce stable dispersions as upon storage there was a tendency of SUL to convert to a more stable crystalline form evident from DSC thermograms. Based on solid state stability data, formulations containing SUL + PPP + PEG1000 and SUL + HPMC + PEG1000 were stable under tested conditions. Also the dynamic vapor sorption analysis suggested better stability of these two formulations with both the formulations absorbing not more than 5% by weight when exposed to 80% relative humidity. The claim that hot melt mixing can be used as a screening technique prior to hot melt extrusion was validated as the combination of SUL + PPP + PEG1000 as obtained by hot melt mixing was able to produce solid dispersions when subjected to hot melt extrusion using a mini lab extruder as confirmed by DSC and X-ray diffraction results.

## 5. Conclusion

A fast and effective screening technique to develop stable solid dispersions for a poorly soluble drug is successfully developed. The given method of hot melt mixing is easy to use, requires less quantity of drug (advantageous for developing solid dispersions of new chemical entities), and is fairly accurate in predicting the amorphization of the drug in formulation.

## Figures and Tables

**Table 1:** Different polymers and plasticizers along with drug tested by film technique.

Polymer^a^	Plasticizer^b^	Solvent used^c^
GB, LM, SM, PPP, CO, PO, P407, GM, TPGS, HPC	—	Chloroform (25–50 mg/mL)
HPMC	—	Methanol : dichloromethane (2 : 1, 25 mg/mL)
Chitosan	—	Water : glacial acetic acid (2 : 1, 20 mg/mL)
—	PEG1000, PEG1000, TEC, C 40	Soluble/miscible with ethanol and chloroform

^a^GB (glyceryl behenate), LM (lauroyl macrogolglycerides), SM (stearoyl macrogolglycerides), PPP (polyvinyl pyrrolidone-polyvinyl chloride-polyvinyl acetate copolymer), CO (copovidone), PO (povidone), P407 (poloxamer 407), GM (glyceryl monostearate), TPGS (D-alpha-tocopherol polyethylene glycol 1000 succinate), HPC (hydroxypropyl cellulose), HPMC (hydroxypropyl methyl cellulose), TEC (triethyl citrate), and C40 (glycerol polyethylene glycol oxystearate).

^b^Plasticizers are used in the study at two levels of 10% w/w and 20% w/w of polymer.

^c^Values in the parenthesis are the solubility of the polymer in the given solvent.

**Table 2:** Formulations of solid dispersion by hot melt mixing.

Formula	Drug (mg)	PPP (mg)^a^	HPC (mg)^a^	HPMC (mg)^a^	PEG1000 (mg)^b^	TEC (mg)^b^	% Yield^c^	% Drug content
A	20	100	—	—	20	—	89.07	100.46
B	20	100	—	—	—	20	77.18	99.66
C	20	—	100	—	20	—	98.90	108.88
D	20	—	100	—	—	20	93.86	98.64
E	20	—	—	100	20	—	98.51	94.06
F	20	—	—	100	—	20	96.64	103.12

^a^The polymers selected for hot melt mixing based on initial screening by film technique.

^b^The plasticizers selected for hot melt mixing at a concentration of 20% w/w of polymer concentration.

^c^The % yield was determined by dividing the total weight of formulation obtained after hot melt mixing process with the total weight of polymer, plasticizer, and SUL used to prepare the formulation.

^d^% content was determined by extracting SUL in suitable solvent and analyzing the SUL content by UV spectroscopy at 327 nm.
